# Supplementary material for: Tumor budding score predicts lymph node status in oral tongue squamous cell carcinoma and should be included in the pathology report
Source: PLoS One. 2020 Sep 25;15(9):e0239783. doi: 10.1371/journal.pone.0239783 (PMC7518591; doi:10.1371/journal.pone.0239783)
Supplement: Attachment — Submitted filename: Response to reviewers 12.07.20.docx [file pone.0239783.s002.docx]

**Response to reviewers**

Thank you for reviewing our manuscript “Tumor budding score predicts lymph node status in oral tongue squamous cell carcinoma and should be included in the pathology report” PONE-D-20-15815, and allowing us to submit a revised version. We appreciate the constructive comments made by the reviewers, which we have answered as specified below, point- by point. We hope you find the changes appropriate.

**Responses to comments made by** **the academic editor:**

1. PLOS ONE’s style requirements

*We have ensured that the manuscript meets the PLOS ONE’s style requirements.*

2. Please ensure that potentially limitations have been discussed

*We have discussed the limitations of the study in the discussion. This includes the study being smaller than some previous studies.*

3. Acknowledgement and Funding statement

*We have withdrawn the text about funding from the Acknowledgements section. Instead, we would like to change the funding statement to: “Inger-Heidi Bjerkli has received financial support for this work from Helse Nord (Nonprofit organization). The funder had no role in planning or execution of the study, nor in interpretation of results or writing of the manuscript.”*

**Responses to comments made by reviewer 1:**

1. Increasing evidences showed that tumor budding, DOI and combination of tumor budding with DOI are correlated with lymph node metastasis in oral tongue squamous cell carcinoma (OTSCC) and early stage OTSCC. It does not add new knowledge to the existing literature.

*We agree that several studies have assessed the correlation between tumor budding, DOI and lymph node metastases previously. For prognostic markers to be included in clinical practice, their relevance must be validated in several independent patient cohorts. Thus, we believe the present study is important although other studies have reported similar results previously. Furthermore, the correlation with lymph node metastases is only one of several results reported in our manuscript.*

2. In page 8, line 146-150, the authors claimed that they combined tumor budding and DOI, but the following description was tumor thickness. Authors should state this clearly since the DOI is not same with tumor thickness.

*We agree that the text is not precisely formulated. Almangush uses the term “tumor invasion” in the work with tumor budding and DOI (reference 14), and we have in our text used the term tumor thickness. We have indeed measured depth of invasion, and have corrected the revised manuscript accordingly.*

3. As shown in Table 4, HRs values are beyond 95%CI. Authors should carefully check the statistical analysis.

*We thank the reviewer for noting this mistake, which was due to inconsistent use of reference category. We have now corrected this in the manuscript.*

4. Several studies showed that TB was a prognostic marker for early stage OTSCC, which was contradictory with the results in this study. The author should discuss it in the part of discussion.

*We agree that our results are not in line with several previous studies finding tumor budding to be a prognostic marker in low-stage OTSCC. The study by Amangush et al. (2015) and Xie et al (2015) included more patients with low-stage disease than our study did. Although we believe that our cohort is of reasonable size, our study may have been underpowered when we separate it into sub-groups. We have included this in the discussion and noted it to be a limitation of our study. Furthermore, the proportion of tumors with high-grade budding was lower in our study compared to the previous studies by Almangush and Xie. Few cases in the high-grade budding group could also inflict on the prognostic value. We have discussed this in the Discussion part.*

**Responses to comments made by Reviewer 2**

MAJOR comments:

1. Authors could consider writing a table which includes the current criteria for grading T and add their suggested inclusions in italics including the strict criteria of measuring budding.

*We have considered writing a table including the current criteria for grading T as well as tumor budding. However, as we have not proposed to include tumor budding into a new TNM we find this difficult. We may have misunderstood the reviewer, and we are willing to look into this again if the reviewer can elaborate on this comment somewhat more.*

2. Previous references that support including TB in T-grading using the high (>=5) or low (<5) buds/field. The authors here used the two and three tiered systems. They should comment based on their results which one seems more appropriate, and add it to the above mentioned Table.

*None of the TB scoring systems were independent prognosticators of survival in multivariate analyses in our study. The correlation to lymph node metastases was stronger for the TB 3-tier system than for the 2-tier system. The HR in univariate survival analyses was larger for the TB 2-tier system than for the 3-tier system, but with a larger CI and higher p-value. Taken together these results suggest that the TB 3-tier system has better prognostic power than the 2-tier system, but our results do not allow definite conclusions to be drawn. We have included a paragraph discussing this in the Discussion part of the manuscript.*

3. Reference 17 refers to colorectal carcinoma TB? This seems an inappropriate reference in citing OTSCC

*We have removed this reference in the revised manuscript.*

MINOR comments:

1. Abstract - a large number of grammatical errors. Please revise.

*We agree and apologize, and have revised the abstract as suggested.*

2. Page 2 background: “the majority of oral cavity arises in the oral tongue”. Should be “the majority of oral cavity cancers arise in the oral tongue”.
   “where of depth of invasion” should be “where depth of invasion”
3. Methods: Page 2 Line 31 “diagnosed with primary oral tongue squamous cell were” should be “diagnosed with primary oral tongue squamous cell carcinoma were”
4. Page 16 line 270: “The arctic University of Norway”. Should be capitalized
5. Page 5 line 81-82 “those who remains” should be “those who remain”

*We appreciate the linguistic corrections, and have corrected as suggested in the revised manuscript.*
