# Supplementary material for: Tumor budding score predicts lymph node status in oral tongue squamous cell carcinoma and should be included in the pathology report
Source: PLoS One. 2020 Sep 25;15(9):e0239783. doi: 10.1371/journal.pone.0239783 (PMC7518591; doi:10.1371/journal.pone.0239783)
Supplement: Attachment — Submitted filename: Response to reviewers 08092020.docx [file pone.0239783.s003.docx]

**Response to reviewers**

Thank you for reviewing our manuscript “Tumor budding score predicts lymph node status in oral tongue squamous cell carcinoma and should be included in the pathology report” PONE-D-20-15815R1, and allowing us to submit a second revision of the manuscript. We appreciate the constructive comments made by the reviewers, which we have answered as specified below, point- by point. We hope you find the changes made appropriate.

**Responses to comments made by** **the academic editor:**

We have no laboratory protocols as we have only done statisitcal analyses from our database.

**Responses to comments made by reviewer 3:**

1. All results were considered significant -> The significance level for the hypothesis tests was 0.05.

*In the abstract and in the method part, under statistics (line 167):
All results were considered significant if p ≤ 0.05 has been changed to: The significance level for the hypothesis test was 0.05.*

2. Add citation for hypothesis testing: <https://www.mdpi.com/2504-4990/1/3/54>

*The citation for hypothesis testing has been added to the materials and method section (statistical analyses).*

3. Table 1 is unclear.

   *We agree that table 1 is unclear and have extended the heading. We hope that it now is more to the point:*

   *Shift in T-classification; pTold in the first column, the pT8th in the second. Coincident numbers indicates no changes in T-classification while discrepancy indicates a shift from a lower to a higher classification, or from a higher to lower (only two cases).* The third column indicates the number of cases for each of the alternative
4. Correlation analysis was not explained explicit enough, and it was not clear which vectors were in the correlation.

   *We agree that this was not explained explicit enough, and have rewritten the first sentence in the paragraph where the vectors are better presented:*

   *Correlation analyses were performed on the whole cohort to evaluate if there was a statistical relationship between tumor budding (2 and 3 tier) and depth of invasion, T-classification (old and new) or lymph node status (N).*
5. It is not clear for which calculations bootstrap has been performed.

   *We agree that this was not specified for each table. Under the material and method section (statistical analyses), we have now specified that we used bootstrapping when performing Spearman correlation analyses (table 2 and 3), and when applying Cox regression. This is in table 4 and 5.*
6. Univariate survival analysis: Please add Kaplan Meier Curves and discuss them in the text.

   *We have added Kaplan Meier curves (figure 2) for budding, and have discussed this in the text (discussion part):

   The* *Kaplan Meier curves also demonstrates that the high budding group according to the 3-tire system has particularly poor survival, suggesting that patient in this group may require more extensive treatment and a closer follow-up than patients with low-budding tumors.*
7. The multivariate section is unclear.

   *We agree that this section could be clearer. We have rewritten parts of the section, and hope it is now more to the point. We have attached a manuscript file with all changes labeled (track changes) and one without mark-ups.*

Best regards

Sonja Steigen, MD, PhD

Tromsø 08.09.2020
